# Influence of Thermally-Accelerated Aging on the Dynamic Mechanical Properties of HTPB Coating and Crosslinking Density-Modified Model for the Payne Effect

**DOI:** 10.3390/polym12020403

**Published:** 2020-02-11

**Authors:** Yongqiang Du, Jian Zheng, Guibo Yu

**Affiliations:** Shijiazhuang Campus, Army Engineering University, Shijiazhuang 050003, China; dyqangus@163.com (Y.D.); zhangxiaopaper@163.com (G.Y.)

**Keywords:** HTPB coating, thermal accelerated aging, dynamic mechanical property, Payne effect, crosslinking density modified model

## Abstract

Hydroxyl terminated polybutadiene (HTPB) coating is widely used in a solid rocket motor, but an aging phenomenon exists during long-term storage, which causes irreversible damage to the performance of this HTPB coating. In order to study the effect of aging on the dynamic mechanical properties of the HTPB coating, the thermally-accelerated aging test was carried out. The variation of maximum elongation and crosslinking density with aging time was obtained, and a good linear relationship between maximum elongation and crosslinking density was found by correlation analysis. The changing regularity of dynamic mechanical properties with aging time was analyzed. It was found that with the increase of aging time, *T_g_* of HTPB coating increased, *T_α_*, *tan β* and *tan α* decreased, and the functional relationships between the loss factor parameters and crosslinking density were constructed. The storage modulus and loss modulus of HTPB coating increased with the increase of aging time, and decreased with the increase of pre-strain. The aging enhanced the Payne effect of HTPB coating, while the pre-strain had a weakening effect. In view of the Payne effect of HTPB coating, the crosslinking density was introduced into Kraus model as aging evaluation parameter, and the crosslinking density modified models with and without pre-strain were established. The proposed models can effectively solve the problem that the Kraus model has a poor fitting effect under the condition of small strain (generally less than 1%) and on the loss modulus, which have improved the correlations between the fitting results and the test results.

## 1. Introduction

A polyurethane network system based on hydroxyl terminated polybutadiene (HTPB) has been widely used in composite solid propellant, adhesive, sealant, coating, and so on, because of its convenient processing and good mechanical properties [1]. HTPB coating is mainly used in the solid rocket motor; however, the aging phenomenon exists during the storage process, which will directly affect the service life of an HTPB coating.

As a molecular structure parameter, crosslinking density affects or even determines the mechanical properties of the material, such as tensile strength, elongation, modulus, compression deformation, swelling rate, hardness, etc. Therefore, compared with the macro-mechanical properties (such as elongation, strength, etc.), crosslinking density is more suitable for being the aging evaluation parameter of HTPB coating [2,3]. Zhao et al. [4] tested the crosslinking density of the material using the method of ^1^H NMR (nuclear magnetic resonance), and analyzed the influence of the crosslinking density on the macro-mechanical properties. 

It was found that with the increase of crosslinking density, the hardness and modulus of the material increased linearly, while the elongation at break decreased linearly. Katarzyna et al. [5] found that the existence of crosslinking resulted in the increase of the glass transition temperature *T_g_*. Actually, the linear relationship equation between *T_g_* and crosslinking density was derived in the study of Fox and Loshaek [6]. Although the linear equation was not completely consistent with some later results [7,8,9], they also came to the same conclusion that the crosslinking can increase the glass transition temperature of the polymer. In the study of Celina et al. [10], it was found that the crosslinking density of HTPB elastomers increased with the aging time. The mainly aging reaction was oxidation crosslinking, and the change of crosslinking density affected the mechanical properties of the material. Byungwoo et al. [11] also got the same conclusion. The test results demonstrated that the crosslinking density increased with the increase of aging temperature and aging time, and the crosslinking changed with the change of aging condition. The functional relationship between strain energy density and crosslinking density was established.

Dynamic mechanical analysis (DMA) is an important tool to study the dynamic mechanical properties of HTPB coating [12]. With the increase of strain amplitude, the typical Payne effect appears in the dynamic mechanical properties of the material [13,14]; that is, the storage modulus decreases with the increase of strain amplitude, while the loss modulus increases first, and then decreases. The famous Kraus model was first proposed by Kraus [15] to describe the Payne effect of materials. Based on the study of Kraus, the loss modulus model was modified by Ulmer [16]. Cho et al. [17] established the viscoelastic constitutive equation based on comprehensively considering the Payne effect, pre-strain and frequency of the material. Wu [18] found that under large strain (more than 10%), there was a deviation between the fitting results of the Kraus model and the test results, and the fitting effect was worse than that under a strain less than 10%. The crosslinking density can be used to quantitatively characterize the change of the molecular structure of materials, but it is rarely reported as to introducing the crosslinking density into the modeling and analysis for the Payne effect of materials.

HTPB coating will be affected by pre-strain during storage, so the dynamic mechanical properties at different aging stages under pre-strain gradually attract people’s attention. Azoug et al. [19] studied the nonlinear, viscoelastic behavior of the propellant under complex loading conditions by means of pre-strained dynamic mechanical analysis. The results demonstrated that the storage modulus and relaxation modulus of the propellant increased with the increase of pre-strain. Thorin et al. [20] modified the traditional generalized Maxwell model by introducing the pre-strain into the model, which effectively described the storage modulus and loss modulus of the HTPB propellant under pre-strain. Kergourlay et al. [21,22] analyzed the constitutive relation of the material under pre-strain, and established the corresponding constitutive model. However, there are few reports on the dynamic mechanical properties of HTPB coating under pre-strain.

In this paper, the thermal accelerated aging test of HTPB coating was carried out, and the correlation between the maximum elongation and the crosslinking density of HTPB coating was analyzed. The changing regularity of the loss factor with the aging time was obtained, and the functional relationship between the loss factor parameters and the crosslinking density was constructed. The effects of thermal accelerated aging and pre-strain on the dynamic mechanical properties and Payne effect of HTPB coating were analyzed. The crosslinking density modified models of HTPB coating with and without pre-strain were established by using the crosslinking density as the aging evaluation parameter, and the correlations between the model fitting results and the test results were analyzed.

## 2. Materials and Methods

### 2.1. Materials

**Figure 1:**
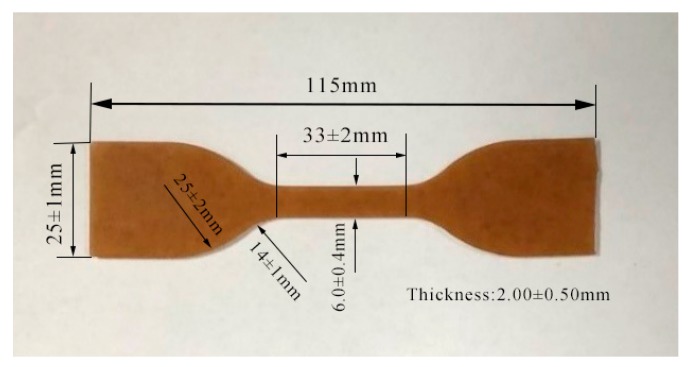
Dimension of the dumbbell-shaped sample of the hydroxyl terminated polybutadiene (HTPB) coating.

The HTPB coatings used in this paper were provided by the State’s No. 845 factory in Xi’an, China, which main components are HTPB and TDI (toluene diisocyanate) [23,24]. According to the proportional parameter provided by the No. 845 factory, the HTPB was used as adhesive, the TDI was used as a curing agent, the diisooctyl sebacate was used as the plasticizer, the zinc oxide was used as the reinforcing, active and vulcanizing agent, the silicon dioxide was used to improve the performance of ablation and corrosion resistance, the molecular sieve was used as the catalytic agent and adsorbent. After curing and shaping in a mold at 70 °C for 5 days, during which process the materials were crosslinked, the HTPB coatings were cut into dumbbell-shaped samples according to the standard I in QJ 916-85 (Space Agency 708, Beijing, China) before tests, as shown in Figure 1.

### 2.2. Thermal Accelerated Aging Test

Thermal accelerated test is mostly used to evaluate the aging performance and storage life of polymer materials [25,26]. The thermal accelerated aging test of the HTPB coating was carried out in the electrothermal oil bath thermostat (Shanghai Experimental Istrument Factory Co., Ltd., Shanghai, China), with the test temperature of 70 °C and the temperature fluctuation range of ±1 °C. The HTPB coatings were taken out at 0 d, 6 d, 12 d, 20 d, 30 d and 40 d, respectively, and then cooled naturally in vacuum drying oven for 24 h before other mechanical properties tests.

### 2.3. Uniaxial Tensile Test

The uniaxial tensile test of HTPB coating was carried out in an Instron 5982 material testing machine (Instron, Boston, MA, USA.). The test temperature was controlled at 25 ± 2 °C, and the tensile rate of the testing machine was 100 mm/min. The maximum elongation of HTPB coatings aged for different time were tested respectively.

### 2.4. Low-field ^1^H NMR Crosslinking Density Test

The crosslinking density of HTPB coating was tested in the VTMR20-010V-T (Shanghai Niumag Electronic Technology Co., Ltd., Shanghai, China) low-field ^1^H NMR relaxometer, with a magnet strength of 0.5 T, coil diameter of 10 mm, resonance frequency of 22.35 MHz, and magnet temperature of 32.00 °C. The Carr-Purcell-Meiboom-Gill (CPMG) (Shanghai Niumag Electronic Technology Co., Ltd., Shanghai, China) sequence was used in the crosslinking density test. The HTPB coating was cut into rectangular strips of 10 mm × 6 mm × 2 mm and put into the tube with an outer diameter of 10 mm. The instrument temperature was set to 90 °C (120 °C above the *T_g_* of the HTPB coating). Before the test, the HTPB coating was kept at 90 °C for 30 min.

### 2.5. Dynamic Mechanical Property Test

The dynamic mechanical properties of HTPB coatings at different aging stages were tested on DMA Q800 (TA Instruments, New Castle, PA, U.S.A.). The tensile clamp was used in the test. The size of the samples were cut into 15 mm × 6 mm × 2 mm. Before the test, the samples were kept in the respective test environment for 5 min.

(1) Dynamic temperature scanning. The temperature scanning range was −80 °C~80 °C. Liquid nitrogen was used for refrigeration. The heating rate was 3K/min, the strain amplitude was 0.1% and the loading frequency was 5 Hz;
(2) Pre-strain dynamic strain scanning. The test temperature was set at 25 °C, and the loading frequency was 5Hz. The results of finite element analysis demonstrated that the maximum strain of the coating is about 9% [27] during long-term storage. Therefore, the pre-strains were selected as 0%, 3%, 6% and 9%, respectively, and the scanning range of the dynamic strain amplitude was 0.1%~10%.

## 3. Results

### 3.1. Correlation Analysis of Maximum Elongation and Crosslinking Density

#### 3.1.1. Aging Results of Maximum Elongation

**Figure 2:**
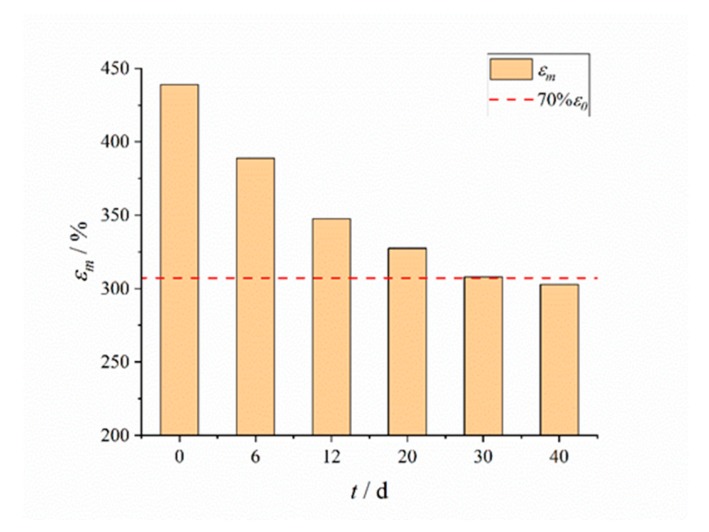
Aging results of maximum elongation of the HTPB coating.

The aging results of the maximum elongation of HTPB coating are shown in Figure 2. The maximum elongation of HTPB coating decreased with the increase of aging time, and the decreasing speed was fast at first, and then slowed down. The change of maximum elongation of HTPB coating was the result of post curing, oxidative crosslinking and degradative chain scission. At the early aging stage, the post curing effect was obvious, which leads to the rapid decrease of the maximum elongation of the HTPB coating. At the middle and later aging stages, the post curing effect was basically complete; there were mainly the interaction of oxidative crosslinking and degradative chain scission. These two effects were basically the same, but the oxidative crosslinking effect was slightly greater than the degradative chain scission effect, hence the maximum elongation continued to decline, but the decline rate tended to slow down.

The red dotted line in Figure 2 indicates the limit of 30% reduction in maximum elongation. In the studies of Zou [28] and Chen [29], the maximum elongation decreased by 30% was taken as the failure criterion of materials. In our test, the maximum elongation of HTPB coating was slightly higher than 70% of the initial maximum elongation at 30 d, and the decrease of the maximum elongation at 40 d had exceeded 30%. It was also found that a small number of microcracks have appeared in the sample after aging for 40 d. It was reasonable to believe that HTPB coating has failed at this time. Therefore, the data of aging for 40 d will not be taken into account in the analysis of subsequent test results. 

#### 3.1.2. Aging Results of Crosslinking Density

**Figure 3:**
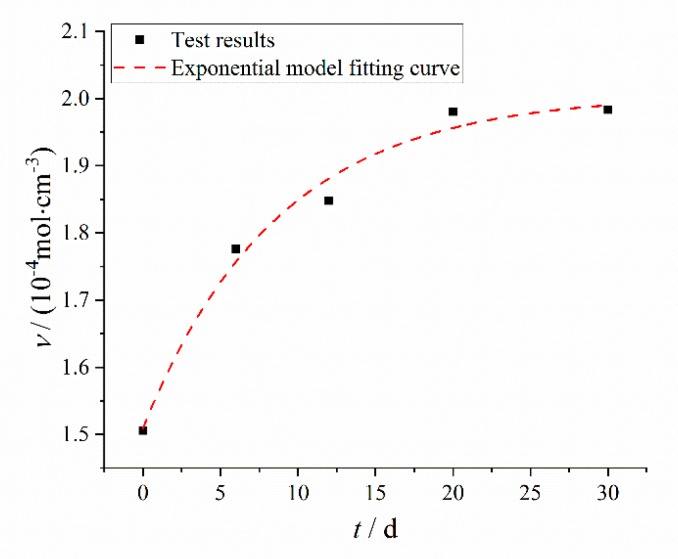
Changing results and fitting curve of crosslinking density with aging time.

The changing results of crosslinking density with aging time is shown in Figure 3. With the increase of aging time, the crosslinking density of HTPB coating increased rapidly at first, and then slowly. The change of the crosslinking density of HTPB coating was the results of post curing, oxidation crosslinking and degradative chain scission [30]. There was the post curing effect of HTPB and TDI at the early aging stage, and the HTPB coating formed a more compact, three-dimensional crosslinking network structure, which limited the movement of the molecular chain and led to the increase of crosslinking density. During the aging process, oxygen attacked HTPB tertiary carbon atoms to form peroxides, which decomposed to form free radicals. On the one hand, the formed free radicals could carry out disproportionation termination to cause chain scission, so as to increase the content of double bond and carbonyl; on the other hand, the generated free radicals could also cause a crosslinking reaction to increase the crosslinking density. At the same time, the formation of free radicals by double bonds on HTPB under the action of oxygen also had the competition of crosslinking and chain scission [11]. 

In the aging process, oxidative crosslinking was slightly greater than degradative chain scission, and the number of crosslinking points between macromolecular networks was increased and the flexibility of molecules was decreased, which limited the intermolecular slip [31,32] and resulted in the slow increase of the crosslinking density of HTPB coating.

There existed an exponential function relationship between crosslinking density and aging time, which was also widely used in the analysis of the thermal accelerated aging test [33,34], as shown in Equation (1):

(1) $$\nu (t)=\nu_{0}+\alpha \exp(-kt)$$

where *k* is the aging reaction rate constant; *t* is the aging time; *ν_0_* and *α* are the constants.

The Levenberg–Marquardt optimization algorithm [35,36] was used for parameters fitting in Equation (1). The fitting results are shown in Table 1 and the fitting curve is shown in Figure 3.

It can be seen from the fitting results that the correlation coefficient between the fitting results of the model and the test results is *R* = 0.9864, that is to say, the exponential model can correctly describe the relationship between the crosslinking density and the aging time.

#### 3.1.3. Correlation Analysis

**Figure 4:**
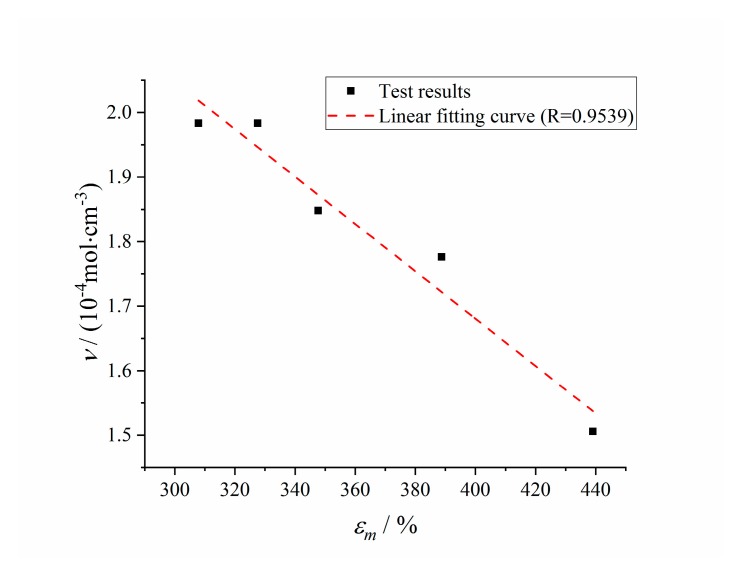
Correlation curve between maximum elongation and crosslinking density.

The correlation between the maximum elongation and the crosslinking density was analyzed, as shown in Figure 4. With the increase of crosslinking density, the restriction of molecular chain movement increased, resulting in the decrease of the maximum elongation [37]. It can be seen from the correlation curve that there is a high linear correlation between the maximum elongation and the crosslinking density of the HTPB coating, and the correlation coefficient *R* = 0.9539.

Crosslinking density is the basic structural parameter to characterize the three-dimensional network of materials. By using crosslinking density as the aging evaluation parameter, on the one hand, it can directly reflect the change of molecular structure of materials [2,3]; On the other hand, the test of crosslinking density is simpler and faster than the maximum elongation, which requires less samples and will not cause damage to the samples.

### 3.2. Analysis and Modeling of Dynamic Mechanical Properties

#### 3.2.1. The Changing Regularity and Modeling Analysis of Loss Factor *tanδ*

**Figure 5:**
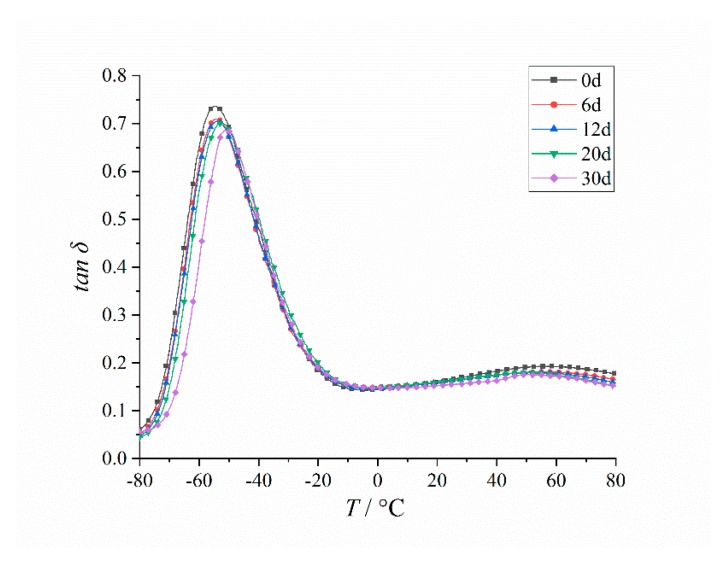
Changing curves of tanδ of the HTPB coating at different aging times.

As shown in Figure 5, the loss factor tanδ curves of HTPB coating at different aging times were obtained using the DMA method:

It can be seen from Figure 5 that at different aging times, the loss factor tanδ of HTPB coating has two peaks, among which the β peak (low temperature peak) is narrow, and the peak height is large, which corresponds to the glass transition temperature *T*_g_ of the HTPB coating. The glass transition temperature *T*_g_ is also one of the most important characteristics of this HTPB coating [38]. The α peak (constant temperature peak) is wide, and the peak height is low. The main reason for the formation of the *α* peak was the polarity difference between the soft segment of butadiene and the hard segment of diisocyanate. When the volume fraction difference between the soft segment and the hard segment was large, the physical crosslinking point formed by the microphase separation was destroyed, and the larger polymer segment could move to form the *α* peak [39].

**Figure 6:**
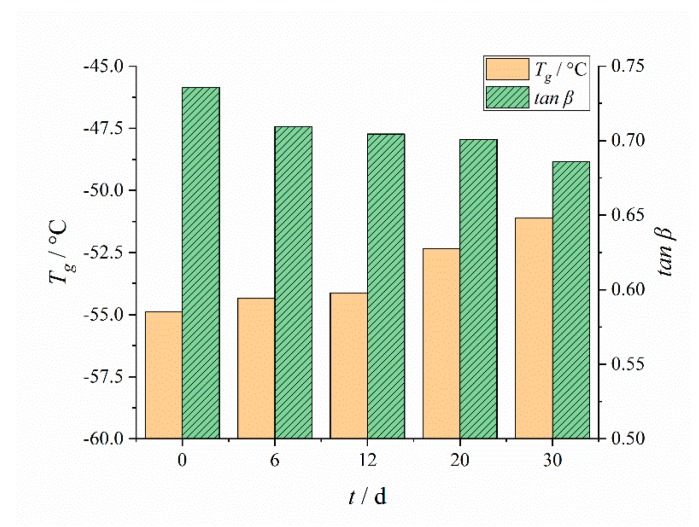
Changing results of *T*_g_ and tanβ with aging time.

**Figure 7:**
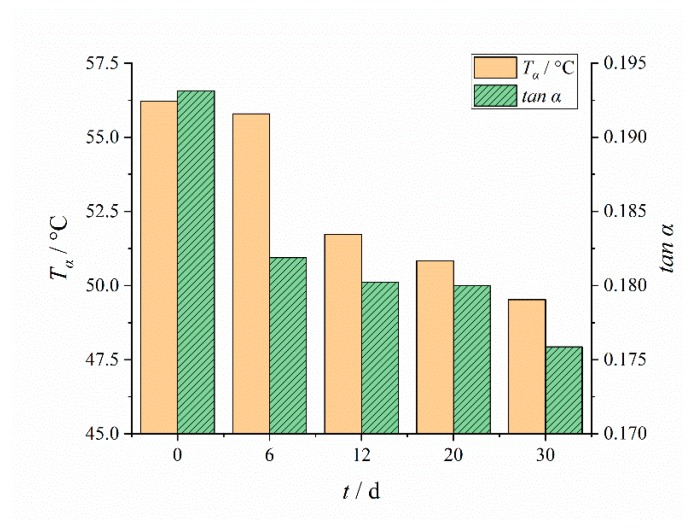
Changing results of *T*_α_ and tanα with aging time.

The temperature (*T*_g_) and peak value (tanβ) corresponding to the β peak and the temperature (*T*_α_) and peak value (tanα) corresponding to the α peak were analyzed, as shown in Figure 6 and Figure 7:

*T*_g_ increased with aging time as can be seen from Figure 6. This was mainly due to the dominant crosslinking reaction in the aging process, which made the material more compact and hard [36,40]. As a kind of topological constraint, crosslinking hindered the chain segment movement. The denser the crosslinking network, the shorter the molecular chain segment between the crosslinks. With the increase of crosslinking density, the movement of polymer segments was more limited, and higher thermal energy was needed to realize the molecular movement of polymer segments. 

The oxidation crosslinking in the aging process made the network structure generate new crosslinking points, so that the volume fraction of the soft and hard segments was almost the same, the microphase separation was less and less, the length and movement range of the polymer segments were limited, and the energy consumption of the movement was also reduced. Therefore, the peak value tanα of the α peak, which represents the mechanical loss, was reduced, and the temperature *T*_α_ corresponding the *α* peak had a decreasing tendency [39].

The exponential models of *T*_g_, *T*_α_, tanβ and tanα with aging time were established and the results are shown in Table 2.

It can be seen from the fitting results that the correlation coefficients between the fitting results and the test results were all greater than 0.9100, which demonstrated that the exponential models can be used to describe the relationship between these four parameters and the aging time. Since the aging test was carried out at a high temperature of 70 °C, and the actual storage temperature is about 25 °C, in order to obtain the actual storage aging time corresponding to the high temperature aging time, it is necessary to convert the high temperature aging time according to the principle of time–temperature equivalence, during which errors will be introduced into the results. In order to solve this problem, and also for the convenience of practical usage, the crosslinking density was used as the characterization parameter to replace the aging time *t*. Solve the inverse function of Equation (1), namely:

(2) $$t=f^{-1}[\nu (t)]$$

That is:

(3) $$t=\frac{1}{k}\ln\frac{\alpha }{\nu -\nu_{0}}$$

Taking Equation (3) into the exponential models (Table 2), the functional relationship between the loss factor parameters and the crosslinking density can be established. In this way, only the crosslinking density of the HTPB coating needs to be tested in the actual storage process, and the relevant parameters of material loss factor can be obtained.

#### 3.2.2. Crosslinking Density Modified Model for Payne Effect

**Figure 8:**
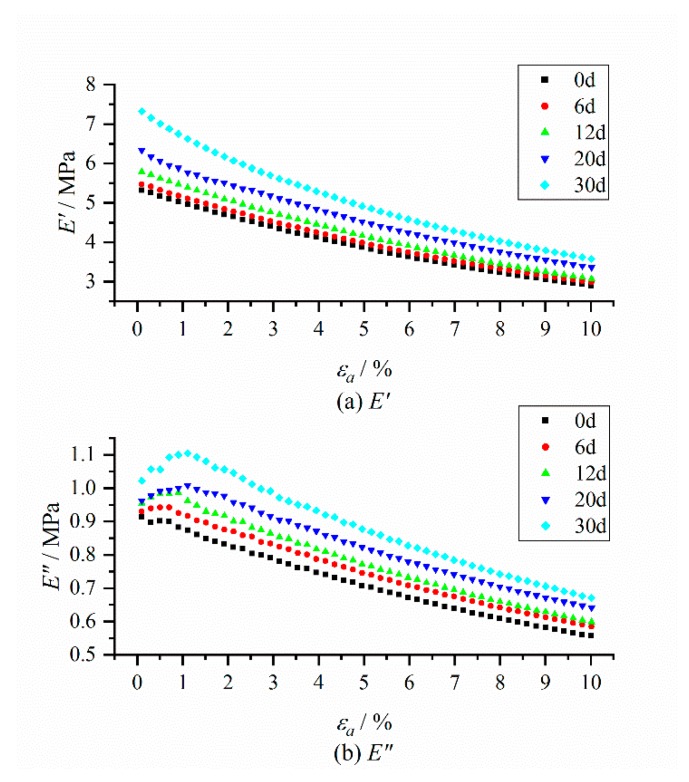
Changing curves of storage modulus and loss modulus with the dynamic strain amplitude at different aging times. (**a**) Storage modulus $E^{\prime }$; (**b**) Loss modulus $E^{\prime \prime }$.

The dynamic strain amplitude scanning results of the storage modulus and loss modulus of HTPB coating at different aging time without pre-strain are shown in Figure 8.

**Figure 9:**
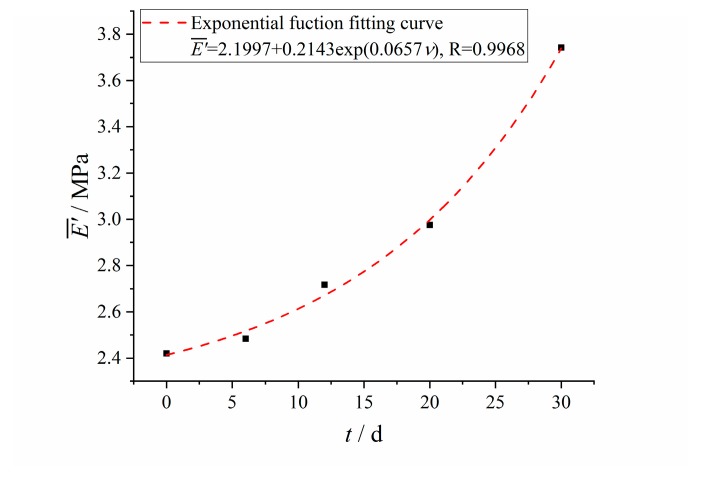
Changing results of the difference of storage modulus with aging time.

The typical Payne effect appeared [13,14] during the dynamic loading process; that is, the storage modulus of HTPB coating at different aging times decreased with the increase of the strain amplitude, while the loss modulus increased first, and then decreased. The strength of the Payne effect can be expressed by the difference of storage modulus ${\bar{E}}^{\prime }$. It can be seen from the test results (Figure 9) that ${\bar{E}}^{\prime }$ increased exponentially with the aging time *t*, which demonstrated that the Payne effect of the material increased with aging time.

**Figure 10:**
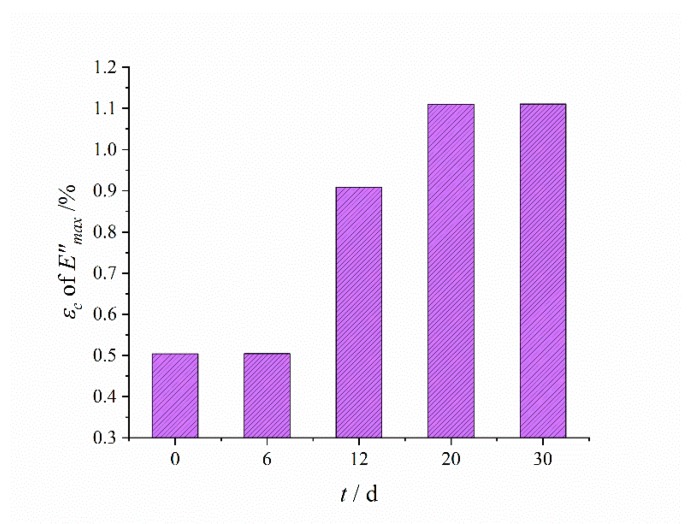
The changing results of strain value $\varepsilon_{c}$ corresponding to the maximum loss modulus ${E^{\prime \prime }}_{max}$ with the aging time.

At the same time, under the same strain amplitude, the storage modulus and loss modulus of the HTPB coating increased with the increase of aging time, which was mainly due to the increase of the crosslinking density of materials during the aging process, and the connection between molecular chains became a more compact three-dimensional network structure, resulting in the restriction of molecular chain movement, thus increasing the modulus of the HTPB coating [41,42]. In addition, it can be seen from the changing curves of the loss modulus that with the increase of aging time, the strain value corresponding to the maximum loss modulus tended to increase, as shown in Figure 10.

The famous Kraus model [15] was first established by Kraus for the Payne effect of materials, that is:

(4) $$E^{\prime }(\varepsilon_{a})=E_{0}^{\prime }-{\bar{E}}^{\prime }+\frac{{\bar{E}}^{\prime }}{1+{\left(\varepsilon_{a}/\varepsilon_{c}\right)}^{2m}}$$

(5) $$E^{\prime \prime }(\varepsilon_{a})=E_{\infty }^{\prime \prime }+\frac{2(E_{\max}^{\prime \prime }-E_{\infty }^{\prime \prime }){\left(\varepsilon_{a}/\varepsilon_{c}\right)}^{m}}{1+{\left(\varepsilon_{a}/\varepsilon_{c}\right)}^{2m}}$$

where ${E^{\prime }}_{0}$ is the storage modulus at small strain amplitude; ${\bar{E}}^{\prime }$ is the difference of storage modulus, and ${\bar{E}}^{\prime }={E^{\prime }}_{0}-{E^{\prime }}_{\infty }$; ${E^{\prime }}_{\infty }\;$ is the storage modulus at large strain amplitude; ${E^{\prime \prime }}_{\max}$ is the maximum loss modulus; $\varepsilon_{c}$ is the strain amplitude corresponding to the maximum loss modulus; *m* is the nonnegative index, which was fixed at 2/3 according to the study of Klüppel and Heinrich [43].

It was found in actual usage that the model fitting results of the loss modulus was not as accurate as that of the storage modulus [44]. In order to solve this problem, Ulmer [16] modified the Kraus model of the loss modulus (Equation (5)), and obtained:

(6) $$E^{\prime \prime }(\varepsilon_{a})=E_{\infty }^{\prime \prime }+\frac{2(E_{\max}^{\prime \prime }-E_{\infty }^{\prime \prime }){\left(\varepsilon_{a}/\varepsilon_{c}\right)}^{m}}{1+{\left(\varepsilon_{a}/\varepsilon_{c}\right)}^{2m}}+\Delta E_{2}^{\prime \prime }\exp(-\varepsilon_{a}/\varepsilon_{2})$$

where $\Delta {E^{\prime \prime }}_{2}$ and $\varepsilon_{2}\;$ are material constants.

The test results were fitted by a Levenberg–Marquardt optimization algorithm [35,36]. It is found that in order to get the most accurate fitting effect, the strain amplitude $\varepsilon_{c}$ of the fitted storage modulus and loss modulus model were not the same, and was not equal to the strain value corresponding to the maximum loss modulus, which is shown in Table 3. At the same time, under the condition of small strain (generally less than 1%), there was a deviation between the model fitting results and the test results, and the fitting effect was slightly worse than that under the condition of the strain greater than 1%.

In order to solve the problems above, and for the convenience of practical test operation, the molecular structure parameter crosslinking density was introduced into the Kraus model, referring to the modified method of the Kraus model by Ulmer [16]. Compared with the $\varepsilon_{c}$, the test of the crosslinking density is more convenient, and it will not cause damage to the sample. The test results of the crosslinking density are more stable and more representative of the change of molecular structure. The crosslinking density modified model is shown below:

(7) $$E^{\prime }(\varepsilon_{a})={E^{\prime }}_{0}-{\bar{E}}^{\prime }+\frac{{\bar{E}}^{\prime }}{1+{\left[\nu (t)\exp(\varepsilon_{a}/\varepsilon_{1})\right]}^{2m}}$$

(8) $$E^{\prime \prime }(\varepsilon_{a})=E_{\infty }^{\prime \prime }+\frac{2(E_{\max}^{\prime \prime }-E_{\infty }^{\prime \prime }){\left\{\varepsilon_{a}/[\nu (t)\exp(\varepsilon_{a}/\varepsilon_{1})]\right\}}^{m}}{1+{\left\{\varepsilon_{a}/[\nu (t)\exp(\varepsilon_{a}/\varepsilon_{1})]\right\}}^{2m}}+\Delta E_{2}^{\prime \prime }\exp(-\varepsilon_{a}/\varepsilon_{2})$$

where ν(t) is the crosslinking density at aging time *t*; $\varepsilon_{1}$ and $\varepsilon_{2}$ are material constants.

**Figure 11:**
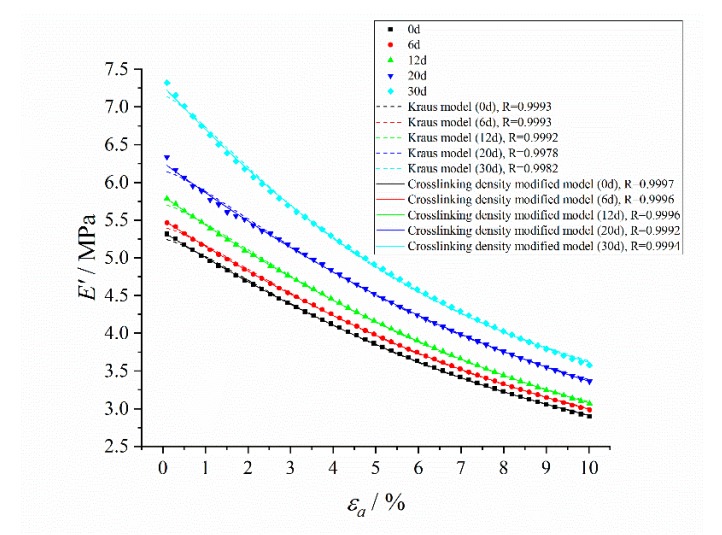
The storage modulus fitting curves of the crosslinking density modified model and Kraus model.

**Figure 12:**
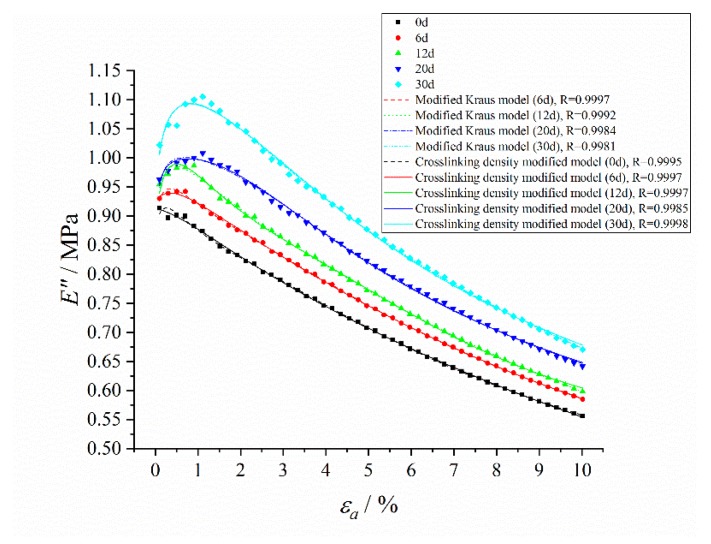
The loss modulus fitting curves of the crosslinking density modified model and modified Kraus model.

The storage modulus fitting curves of the crosslinking density modified model and Kraus model are shown in Figure 11. It can be seen that the crosslinking density modified model effectively improved the fitting effect under small strain (less than 1%), and improved the correlations between the model fitting results and test results compared with the Kraus model. The loss modulus fitting curves of the crosslinking density modified model and modified Kraus model are shown in Figure 12. The crosslinking density modified model can describe the loss modulus accurately, and further improved the correlations compared with the modified Kraus model.

#### 3.2.3. Pre-strain Crosslinking Density Modified Model for Payne Effect

Under different pre-strain conditions, the storage modulus and loss modulus of HTPB coating still maintained the typical Payne effect. Under the same strain amplitude, the storage modulus and loss modulus of HTPB coating decreased with the increase of pre-strain, which was mainly because of the fact that the existence of pre-strain will destroy the micromolecular chain structure and reduce the crosslinking density of the material [19,45].

**Figure 13:**
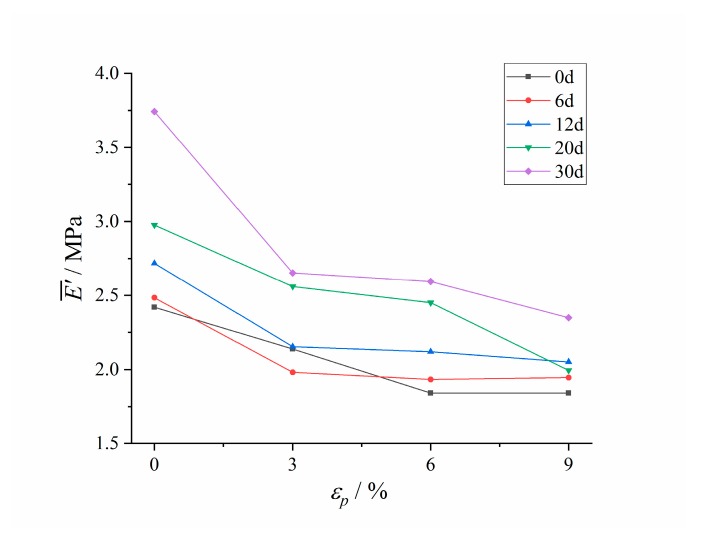
The changing curves of the difference of storage modulus with pre-strain.

The difference of storage modulus ${\bar{E}}^{\prime }$ under different pre-strains were analyzed, as shown in Figure 13.

At different aging stages, the difference of the storage modulus of HTPB coating decreased with the increase of pre-strain, indicating that the Payne effect of HTPB coating weakened gradually with the increase of pre-strain.

**Figure 14:**
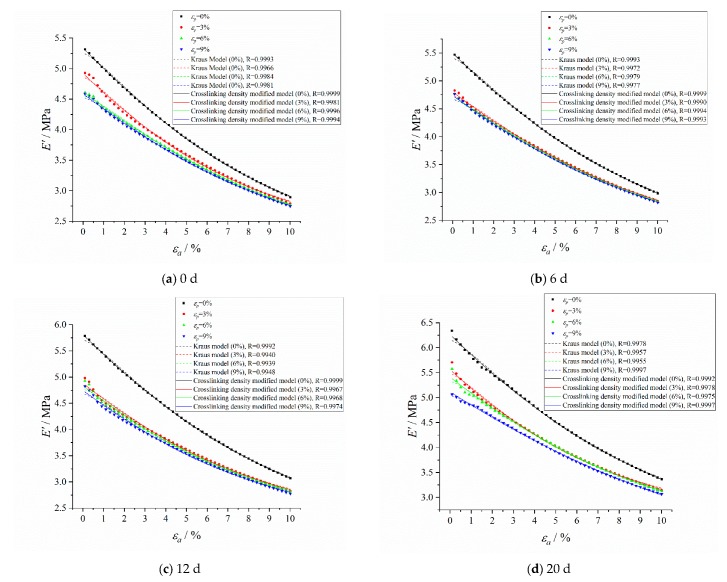

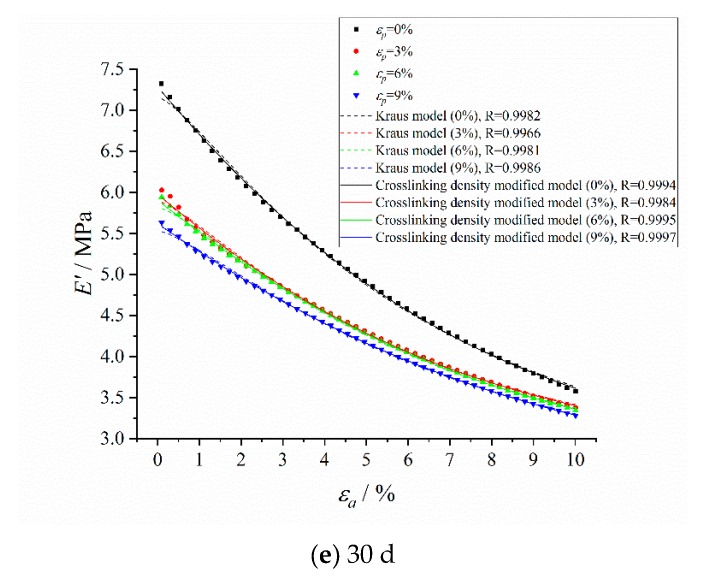
The storage modulus fitting curves of the crosslinking density modified model and Kraus model under pre-strain. (**a**) Aged for 0 d; (**b**) Aged for 6 d; (**c**) Aged for 12 d; (**d**) Aged for 20 d; (**e**) Aged for 30 d.

The crosslinking density modified model and the Kraus model were used to fit the storage modulus of HTPB coating under different pre-strains, and the fitting curves are shown in Figure 14.

It can be seen from the fitting curves that the crosslinking density modified model improved the correlation between model fitting results and test results, and the correlation coefficients were all greater than 0.9967. Meanwhile, the crosslinking density modified model still had a high fitting effect under small strain, which could be used to accurately describe the storage modulus under pre-strain conditions.

The existence of pre-strain will increase the loss of the HTPB coating, and there is stress relaxation in the loading pre-strain process. In order to make the model more accurate, the relaxation factor was introduced into the crosslinking density modified model of the loss modulus, and the pre-strain crosslinking density modified model of the loss modulus was obtained:

(9) $$E^{\prime \prime }(\varepsilon_{a})=E_{\infty }^{\prime \prime }+\frac{2(E_{\max}^{\prime \prime }-E_{\infty }^{\prime \prime }){\left\{\varepsilon_{a}/[\nu (t)\exp(\alpha )\exp(\varepsilon_{a}/\varepsilon_{1})]\right\}}^{m}}{1+{\left\{\varepsilon_{a}/[\nu (t)\exp(\alpha )\exp(\varepsilon_{a}/\varepsilon_{1})]\right\}}^{2m}}+\Delta E_{2}^{\prime \prime }\exp(-\varepsilon_{a}/\varepsilon_{2})$$

**Figure 15:**
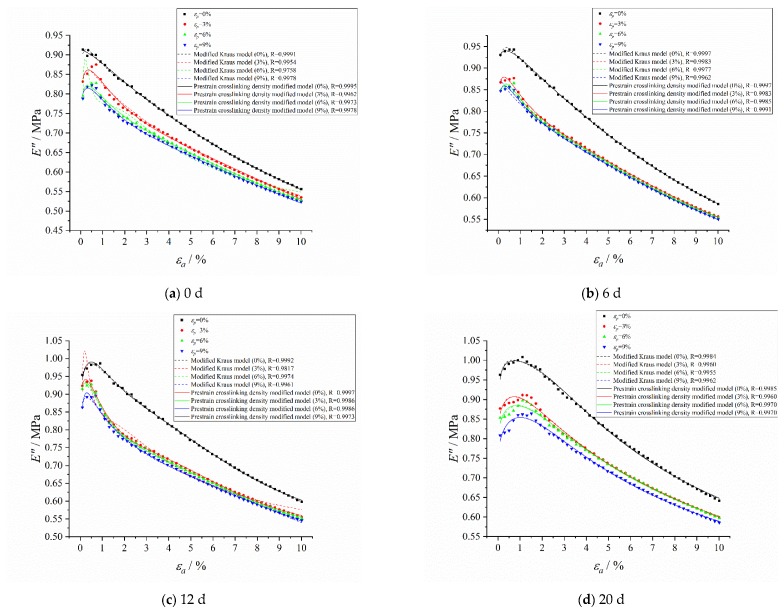

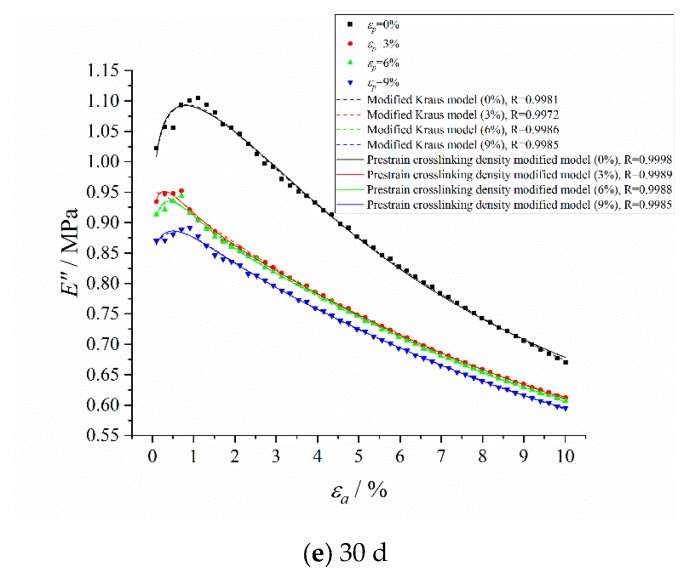
The loss modulus fitting curves of the pre-strain crosslinking density modified model and modified Kraus model under pre-strain. (**a**) Aged for 0 d; (**b**) Aged for 6 d; (**c**) Aged for 12 d; (**d**) Aged for 20 d; (**e**) Aged for 30 d.

The loss modulus of HTPB coating under different pre-strains were fitted by using the pre-strain crosslinking density modified model and the modified Kraus model, respectively, and the results are shown in Figure 15:

It can be seen from the loss modulus fitting curves that the fitting correlations between the fitting results of the pre-strain crosslinking density modified model and test results were all greater than 0.9960, which were better than the modified Kraus model, and also had a higher fitting effect under small strain. Therefore, the proposed model can effectively describe the loss modulus of HTPB coating under pre-strain.

## 4. Conclusions

In this paper, the thermally-accelerated aging test of HTPB coating was carried out, and the maximum elongation, crosslinking density and dynamic mechanical properties of the HTPB coating at different aging stages were tested. By analyzing the changing regularity of mechanical properties, the relevant mechanical models were established with the crosslinking density as the aging evaluation parameter. The following conclusions were obtained:

1. The changing mechanisms of the maximum elongation and crosslinking density of HTPB coating with aging time were analyzed, and the exponential function model of the crosslinking density was established. Through the correlation analysis, it was found that there was a good linear relationship between crosslinking density and maximum elongation, and the correlation coefficient *R* = 0.9539. The crosslinking density can be used as the aging evaluation parameter to analyze the performance of the HTPB coating.
2. With the increase of aging time, the *T*_g_ of the HTPB coating gradually increased, while *T*_α_, tanβ and tanα decreased. The exponential function model of the loss factor parameters (*T*_g_, *T*_α_, tanβ and tanα) with aging time was established. The correlation coefficients between the model fitting results and the test results were all greater than 0.9100. Furthermore, the functional relationships between the loss factor parameters and crosslinking density were constructed.
3. The storage modulus and loss modulus of HTPB coating increased with aging time, and the aging enhanced the Payne effect of the HTPB coating. The crosslinking density was introduced into the Kraus model as the aging evaluation parameter, and the crosslinking density-modified model for the Payne effect was established. The proposed model can solve the problem that the Kraus model has a poor fitting effect under the condition of small strain and on loss modulus, and has improved the correlations between model fitting results and test results.
4. The storage modulus and loss modulus of HTPB coating decreased with the increase of pre-strain, and the existence of pre-strain weakened the Payne effect of HTPB coating. Considering the effect of stress relaxation, a pre-strain crosslinking density modified model for the Payne effect was established. The correlation coefficients between the fitting results and the test results were all greater than 0.9960, which can be effectively used in the description of the Payne effect of an HTPB coating under pre-strain.

## Figures and Tables

**Table 1:** Fitting results of crosslinking density exponential model.

Parameters	*ν_0_*	*k*	*α*	*R*
**Fitting Results**	2.0059	0.1151	−0.4967	0.9864

**Table 2:** Fitting results of loss factor parameters with aging time.

Paremeters	Fitting Equation	Correlation Coefficient *R*
*T*_β_	*T*_β_ = 3.4927exp(0.0252*t*) − 58.4671	0.9728
*T*_α_	*T*_α_ = 10.7218exp(−0.0386*t*) + 45.9629	0.9183
tanβ	tanβ = 0.0474exp(−0.0893*t*) + 0.6867	0.9368
tanα	tanα = 0.0152exp(−0.1846*t*) + 0.1778	0.9480

**Table 3:** Model fitting and test results of $\varepsilon_{c}$.

t/d.	$\varepsilon_{c}$ $\mathrm{of}\;E^{\prime }(\varepsilon_{a})$	$\varepsilon_{c}$ $\mathrm{of}\;E^{\prime \prime }(\varepsilon_{a})$	$\varepsilon_{c}$ $\mathrm{of}\;{E^{\prime \prime }}_{max}$
0	8.1502	2.3803	0.5042
6	8.3056	2.5024	0.5043
12	8.3562	2.9747	0.9089
20	8.4099	2.0871	1.1104
30	6.3903	6.0678	1.1108
